# Assessment of the impact of body mass index on right ventricular systolic and diastolic function in patients with type 2 diabetes mellitus using two-dimensional speckle tracking imaging

**DOI:** 10.3389/fcdhc.2026.1694955

**Published:** 2026-08-26

**Authors:** Wenwen Wang, Yuanyuan Peng, Kun Zhao, Ziwei Zheng, Jiangbo Feng, Chengfang Zou, Yongchun Sun, Bo Lin, Liping Wang, Bing Hu, Shaoling Yang

**Affiliations:** 1 Department of Ultrasound, Shanghai Eighth People’s Hospital, Shanghai, China; 2 Department of Radiology, Shanghai Jiao Tong University Affiliated Sixth People's Hospital, Shanghai, China; 3 Department of Ultrasound, Shanghai Sixth People’s Hospital, Shanghai Jiao Tong University, Shanghai, China

**Keywords:** body mass index, obesity, right ventricular function, two-dimensional speckle tracking imaging, type 2 diabetes mellitus

## Abstract

**Objective:**

To evaluate the impact of different body mass index (BMI) levels on right ventricular (RV) systolic and diastolic function in patients with type 2 diabetes mellitus (T2DM) using two-dimensional speckle tracking imaging (2D-STI), and to provide imaging reference for the early screening of subclinical RV dysfunction in overweight and obese patients with T2DM.

**Method:**

A total of 67 newly diagnosed T2DM patients (disease duration ≤6 months, treatment-naïve) and 61 age-matched healthy controls who presented to our hospital from January 2022 to June 2023 were enrolled. All participants with hypertension, cardiovascular diseases, or bronchopulmonary diseases were excluded. According to T2DM status and BMI levels, the subjects were divided into four groups: non-T2DM normal weight group (BMI 18.5-23.9 kg/m^2^, n=30), non-T2DM overweight/obese group (BMI ≥24.0 kg/m^2^, n=31), T2DM normal weight group (n=33), and T2DM overweight/obese group (n=34). The primary endpoint was RV free wall global longitudinal strain (RVFWS), and the secondary endpoint was the early-to-late diastolic strain rate ratio (SRe/SRa). The sample size was calculated based on a pilot study (expected effect size =1.8, α=0.05, statistical power = 80%), with a minimum required sample size of 28 per group, which was met by the actual enrollment. 2D-STI was performed to measure longitudinal strain at the basal, mid, and apical segments of the RV free wall, and the average value was defined as RVFWS. Early diastolic strain rate (SRe) and late diastolic strain rate (SRa) were recorded, and the SRe/SRa ratio was calculated. Two-way analysis of variance (ANOVA) was used to compare differences among groups, and multiple linear regression analysis was performed to identify independent predictors of RV functional parameters and to test for interaction effects. Continuous variables were expressed as mean ± standard deviation, and *post-hoc* multiple comparisons were conducted using the least significant difference (LSD) method. We integrated interpretations of main and interaction effects to minimize Type I error inflation.

**Results:**

Statistically significant differences in RVFWS and SRe/SRa ratio were observed among the four groups (all P < 0.05). Two-way ANOVA revealed significant interactions between BMI and T2DM for both RVFWS and SRe/SRa ratio (all P < 0.001). Compared with Group A, RVFWS and SRe/SRa decreased stepwise in Group B, Group C and Group D, with the greatest decline observed in Group D. Multiple linear regression demonstrated that BMI was significantly negatively associated with RVFWS and the SRe/SRa ratio in the overall cohort (P < 0.001). Full 95% confidence intervals were calculated and reported for all correlation and regression outcomes.

## Highlights

- 2D-STI can sensitively detect early RV systolic and diastolic dysfunction in patients with T2DM, providing imaging evidence for identifying subclinical myocardial alterations.
- BMI independently correlates with RV dysfunction, and this association is consistent in both T2DM and non-diabetic populations.
- BMI and T2DM exert a statistically significant interactive effect on RV dysfunction, highlighting the necessity of weight management for patients with T2DM.

## Introduction

Type 2 diabetes mellitus (T2DM) is a globally prevalent chronic metabolic disorder, and its prevalence in China continues to rise (1). Cardiovascular complications are the major contributors to all-cause morbidity and mortality among patients with T2DM (2). China bears one of the heaviest global diabetes burdens, and early screening for myocardial dysfunction has attracted increasing domestic attention (3).

While T2DM has been well-documented to be closely associated with structural and functional abnormalities of both the left and right ventricles, existing research has predominantly focused on left ventricular dysfunction, with comparatively less attention devoted to RV alterations. The right ventricle plays a critical role in maintaining pulmonary circulation and global hemodynamic balance, and RV functional abnormalities may serve as a potential early marker of diabetic cardiomyopathy. Mounting evidence suggests that in metabolic diseases including T2DM, RV dysfunction emerges prior to left ventricular lesions and indicates early subclinical myocardial remodeling (4). Accordingly, evaluating RV function in patients with T2DM carries important clinical implications.

Existing data on RV performance in T2DM patients are inconsistent. Some investigations have detected early subclinical systolic and diastolic RV dysfunction among diabetic individuals, while others attribute RV functional changes mainly to disease duration and cumulative metabolic load. Body mass index (BMI), a widely used metric for assessing overweight and obesity, is closely linked to cardiovascular complications in T2DM (5). However, few studies have explored how varying BMI levels affect RV function in patients with T2DM, and early overweight-related changes in RV myocardial mechanics remain poorly clarified (6).

Alterations including insulin resistance, chronic systemic inflammation and myocardial metabolic disturbance accompanied by obesity may contribute to the development of myocardial functional abnormalities in diabetic individuals (7, 8). Elevated BMI is further associated with increased myocardial stiffness, impaired diastolic filling, and heightened heart failure risk (9). Concurrently, bronchopulmonary diseases can induce subclinical RV alterations even in the absence of cardiovascular disease, and thus must be rigorously excluded as a confounder. Elucidating the impact of BMI on RV function in T2DM patients would facilitate improved risk stratification and the development of individualized prevention strategies (10).

Two-dimensional speckle tracking imaging (2D-STI) is a non-invasive echocardiographic modality that enables quantitative assessment of myocardial strain by tracking the motion of intrinsic myocardial acoustic markers (11). Compared with conventional echocardiography, 2D-STI offers superior sensitivity and reproducibility for detecting early myocardial dysfunction, and holds unique advantages in evaluating subtle alterations in RV myocardial mechanics (12).

The primary objectives of this study were threefold: (1) to compare RV systolic and diastolic functional parameters between T2DM patients with varying BMI levels and non-diabetic controls using 2D-STI; (2) to examine the association between BMI and RV functional parameters; and (3) to investigate whether a statistical interaction exists between BMI and T2DM with respect to RV functional decline.

We proposed the following *a priori* hypotheses: (1) patients with T2DM exhibit subclinical RV systolic and diastolic dysfunction at an early disease stage; (2) elevated BMI correlates with RV myocardial abnormalities; and (3) BMI and T2DM exert an interactive and synergistic detrimental effect, such that T2DM patients with overweight/obesity demonstrate the most severe RV functional abnormalities.

Notably, these are only *a priori* hypotheses of this cross-sectional study; we can only analyze statistical associations rather than confirm causal effects between BMI, T2DM and RV dysfunction.

## Methods

This study prospectively enrolled 67 patients with T2DM and 61 age-matched healthy controls who presented to our hospital between January 2022 and June 2023. The diagnosis of T2DM was established according to the 2020 Chinese Guidelines for the Prevention and Treatment of Type 2 Diabetes Mellitus. All participants were screened for bronchopulmonary disease and excluded if positive, as confirmed by chest computed tomography (CT) and pulmonary function testing. Healthy controls had no history of diabetes, coronary artery disease, or hypertension; cardiovascular disease was systematically excluded via medical history review, physical examination, 12 -lead electrocardiography (ECG), and transthoracic echocardiography.

Inclusion criteria for the T2DM group: (1) Newly diagnosed T2DM. Definition of disease duration: Patients with no prior history of diabetes diagnosis and no prior use of antidiabetic medications. Disease duration was calculated as the interval between the date of first T2DM diagnosis by the endocrinology department and the date of the index echocardiographic examination. Only patients with disease duration ≤6 months were enrolled. (2) Meeting any of the following glycemic criteria: fasting plasma glucose ≥ 7.0 mmol/L, random plasma glucose ≥ 11.1 mmol/L, 2-hour oral glucose tolerance test (OGTT) glucose ≥ 11.1 mmol/L, or glycated hemoglobin A1c (HbA1c) ≥6.5%. We documented diabetic symptoms to characterize newly diagnosed T2DM at baseline. Symptom status did not affect eligibility and only served as baseline data irrelevant to our hypotheses. In total, 39 T2DM patients had typical diabetic symptoms while 28 were asymptomatic, assessed via a standardized questionnaire from our endocrinology department. (3) Absence of hypertension (systolic blood pressure < 140 mmHg and diastolic blood pressure < 90 mmHg). Cardiovascular and bronchopulmonary diseases were excluded by combined assessment including medical history review, physical examination, standard 12-lead ECG, transthoracic echocardiography, and chest CT.

The mean glycated hemoglobin (HbA1c) level of patients with type 2 diabetes mellitus (T2DM) in this cohort was 7.0% (7.02% ± 0.56% for Group C, 6.97% ± 0.57%), which was below the HbA1c threshold of 7.5% recommended for initiating hypoglycemic pharmacotherapy in 2020 Chinese Guidelines for the Prevention and Treatment of Type 2 Diabetes Mellitus (13).

Per the above guidelines, newly diagnosed T2DM patients with HbA1c < 7.5% received standardized 3-month lifestyle intervention as first-line treatment, even if they only had mild polydipsia, polyuria or other typical diabetic symptoms. The intervention package included standardized dietary advice, personalized aerobic exercise plans, and monthly weight and glycemic monitoring; no urgent antihyperglycemic drugs were administered at baseline. All T2DM subjects in this study followed this standardized management protocol. Transthoracic echocardiography was uniformly arranged right after the 3-month intervention cycle, so the average drug-free period before enrollment was around 3 months.

Of the 39 patients with typical diabetic symptoms, 36 achieved substantial or full symptom relief within 2–4 weeks of lifestyle adjustment, and the other 3 obtained partial improvement. This clinical response matched the expected disease trajectory of mild hyperglycemia stated in the guidelines.

Three participants still maintained HbA1c > 7.5% after lifestyle modification. Antidiabetic medications were prescribed for them after echocardiography, but their baseline clinical data were retained for this cross-sectional analysis.

Inclusion criteria for healthy controls: (1) No history of diabetes, hypertension, coronary artery disease, bronchopulmonary disease, or other chronic conditions; (2) All laboratory and imaging examinations (echocardiography, chest CT, ECG, pulmonary function tests) within normal reference ranges;(3) BMI between 18.5 and 30.0 kg/m^2^.

**Figure 1:**
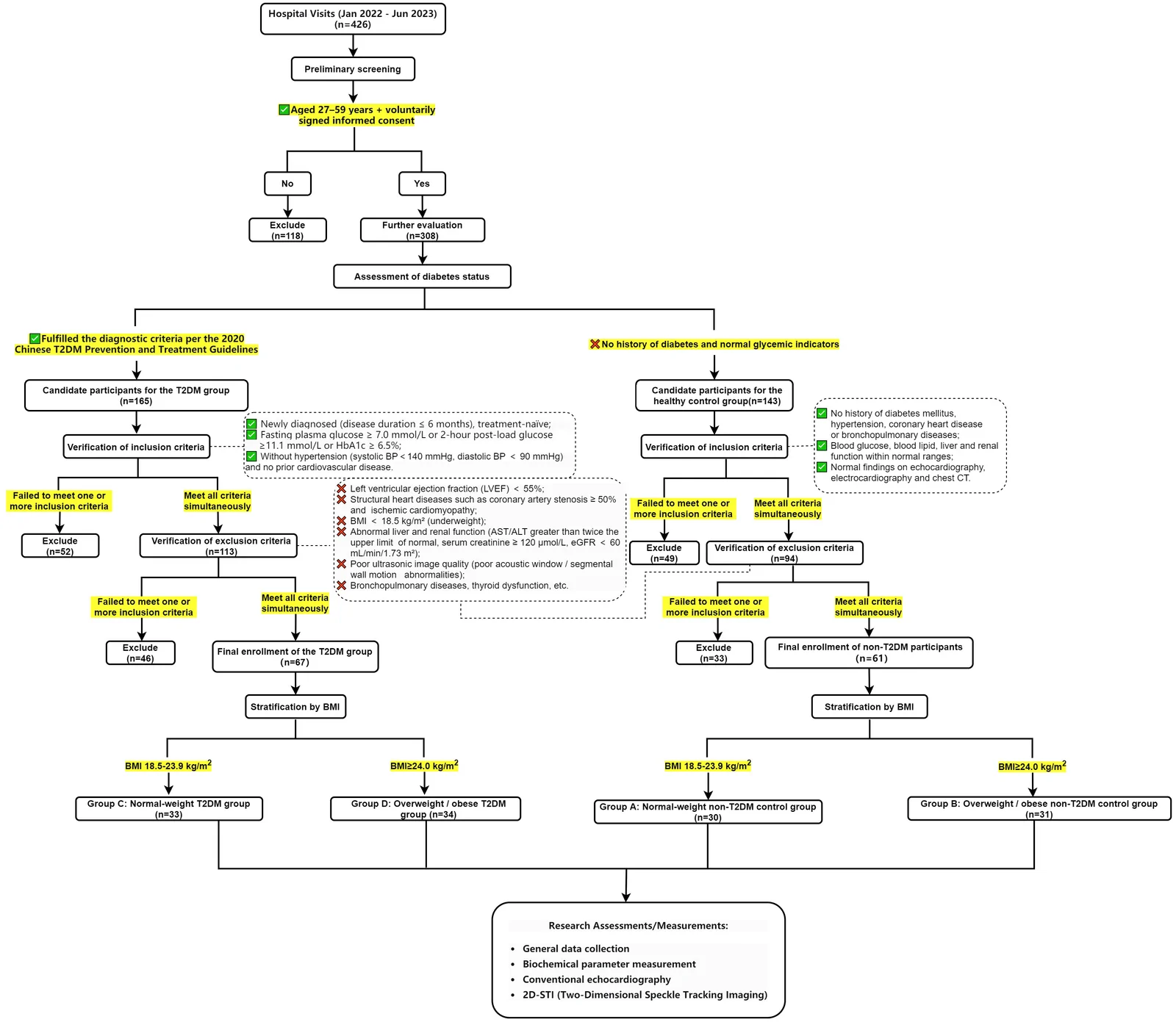
CONSORT flowchart showing participant screening, exclusion and group stratification procedures.

Exclusion criteria: (1) Reduced left ventricular systolic function (left ventricular ejection fraction [LVEF] < 55%); (2) Severe structural heart disease, including coronary artery stenosis ≥50%, ischemic cardiomyopathy, prior myocardial infarction, or congenital heart disease; (3) Underweight (BMI < 18.5 kg/m^2^) with potential malnutrition; (4) Hypertension (systolic blood pressure ≥140 mmHg or diastolic blood pressure ≥90 mmHg), regardless of treatment status; (5) Hepatic or renal dysfunction, defined as aspartate aminotransferase (AST) or alanine aminotransferase (ALT) > 2 times the upper limit of normal, serum creatinine ≥120 μmol/L, or estimated glomerular filtration rate (eGFR) < 60 mL/min/1.73 m^2^; (6) Suboptimal echocardiographic image quality (segmental wall motion abnormalities or poor acoustic window), as determined by criteria from the American Society of Echocardiography; (7) Concomitant conditions potentially affecting cardiac function, including bronchopulmonary disease, thyroid dysfunction, or autoimmune disease (**Figure 1**).

### Study groups

During the enrollment process, baseline characteristics were balanced across groups. In addition to age matching, we ensured comparability in terms of sex, BMI, lipid profiles, and left ventricular ejection fraction (LVEF).

Based on T2DM status and BMI levels (Chinese criteria: normal weight 18.5-23.9 kg/m^2^, overweight/obesity≥24.0 kg/m^2^), the 128 participants were categorized into four groups:

Group A (non-T2DM, normal weight): n = 30, 17 males and 13 females;
Group B (non-T2DM, overweight/obese): n = 31, 15 males and 16 females;
Group C (T2DM, normal weight): n = 33, 16 males and 17 females;
Group D (T2DM, overweight/obese): n = 34, 19 males and 15 females.

This study was approved by the Ethics Committee of Shanghai Eighth People’s Hospital (approval number: 2023-050-10) and was conducted in accordance with the Declaration of Helsinki. All participants provided written informed consent prior to study enrollment.

### Collection of general data and biochemical indicators

Baseline data were collected for all participants, including sex, age, height, body weight, systolic blood pressure (SBP), diastolic blood pressure (DBP), and resting heart rate. After an overnight fast of at least 8 hours, venous blood samples were collected in the morning. Liver function parameters (aspartate aminotransferase [AST], alanine aminotransferase [ALT]), glycemic control indicators (fasting plasma glucose [FPG], glycated hemoglobin A1c [HbA1c]), and lipid profiles (total cholesterol [TC], triglycerides [TG], low-density lipoprotein cholesterol [LDL-C]) were measured. All laboratory tests were performed by the hospital’s central clinical laboratory following standardized protocols.

All hypertensive subjects (including patients receiving antihypertensive treatment) were excluded in strict accordance with the exclusion criteria of this study. The enrolled patients with newly diagnosed T2DM had a disease duration of no more than 6 months and only mild metabolic disorders without vascular remodeling induced by long-term hyperinsulinemia. All participants were outpatients undergoing screening, and none had comorbidities that could elevate blood pressure such as obstructive sleep apnea and severe visceral obesity.

Standardized blood pressure measurement protocols were implemented: subjects rested quietly for 10 minutes in a constant-temperature examination room. Calibrated electronic sphygmomanometers were used to take three measurements at 2 -minute intervals, and the average value of the last two stable readings was recorded. All operations were completed by full-time nurses, eliminating systematic bias toward low blood pressure readings.

All participants were newly diagnosed early T2DM patients with a disease duration ≤ 6 months, who had not developed macrovascular remodeling or sustained volume overload caused by chronic hyperglycemia. In addition, hypertension and masked hypertension were rigorously ruled out through repeated clinic blood pressure measurements and medical history collection at enrollment. Consequently, the systolic blood pressure of the cohort as a whole fell within the low-normal range, consistent with previous reports on populations with early metabolic abnormalities (14).

It should be emphasized that the primary objective of this study was to evaluate the independent effects of BMI and T2DM on RV function, rather than to explore the correlation between blood pressure and ventricular function. Systolic blood pressure was comparable across all groups (P = 0.54) with no statistically significant intergroup differences. Therefore, the relatively low systolic blood pressure levels would not introduce confounding bias to the core intergroup comparative results.

### Acquisition of conventional echocardiographic parameters

All transthoracic echocardiographic (TTE) examinations were performed using a Philips EPIQ7 ultrasound system equipped with an S 5–1 phased array probe (frequency range: 1–5 MHz). Participants were examined in the standard left lateral decubitus position during quiet respiration. A standard precordial lead electrocardiogram (ECG) was recorded simultaneously for accurate timing of the cardiac cycle.

All measurements were independently and blindly performed by two senior echocardiographers certified by the American Society of Echocardiography (ASE) using a standardized dual-operator acquisition protocol. Two senior physicians independently acquired RV functional parameters, and averaged values were adopted to reduce inter-observer variability and boost reproducibility. Pulmonary artery systolic pressure (PASP) was estimated using the tricuspid regurgitation method: PASP = tricuspid regurgitation pressure gradient (4V ^2^) + right atrial pressure. Right atrial pressure was semi-quantitatively assessed based on the inferior vena cava (IVC) diameter and inspiratory collapse rate.

### Acquisition and analysis of 2D-STI parameters

RV-focused apical four-chamber cine loops were acquired and stored in DICOM format. The frame rate of image acquisition ranged from 50 to 70 Hz, with a mean value of 62.4 ± 5.8 Hz, meeting the minimum frame rate requirement (≥50 Hz) recommended by the American Society of Echocardiography (ASE) for RV strain analysis. Intraobserver and interobserver reproducibility tests revealed excellent consistency for RVSRe and RVSRa measurements, with all intraclass correlation coefficients (ICCs) > 0.90 and coefficients of variation (CVs) < 5%, which verified that this frame range can reliably capture peak diastolic strain values without obvious peak underestimation. As acknowledged in the Limitations section of this manuscript, our imaging protocol only set 50 Hz as the minimum frame threshold. Frame rates higher than 70 Hz can further improve the temporal resolution of rapid diastolic strain signals. Accordingly, higher-frame-rate acquisition protocols will be prioritized in future research to minimize the risk of underestimated diastolic strain peaks. Analysis was performed using QLAB software (version 13.0, Philips Healthcare) with the AutoStrain RV module. The RV endocardial border was manually traced at end-systole, and the software automatically divided the RV wall into 6 segments. The width of the region of interest (ROI) was manually adjusted as needed. Given the potential impact of left ventricular contraction on the interventricular septum, only the RV free wall was analyzed in this study.

Longitudinal strain of the basal (RVLSbas), mid (RVLSmid), and apical (RVLSapi) segments of the RV free wall was measured, and the average value was used to calculate RVFWS (RVFWS = ∑RVLS/3). Peak early diastolic strain rate (RVSRe) and peak late diastolic strain rate (RVSRa) were recorded, and the SRe/SRa ratio was calculated.

To assess intra-observer and inter-observer variability, repeated measurements were independently performed by the same operator and a second operator after an interval of 2 weeks.

### Statistical analysis

All statistical analyses were performed using SPSS version 26.0 (IBM Corp., Armonk, NY, USA). Continuous variables were tested for normality using the Shapiro-Wilk test and were expressed as mean ± standard deviation. Categorical variables were expressed as frequencies (percentages) and compared using the chi-square (χ^2^) test. The specific analytical methods were as follows:

1. Baseline comparison among groups: One-way analysis of variance (ANOVA) was used for continuous variables, and the chi-square test was used for categorical variables.
2. Main effects and interaction analysis: Two-way ANOVA was performed to assess the main effects of BMI (normal weight vs. overweight/obese) and T2DM (present vs. absent) on RV functional parameters, as well as their interaction effect.
3. *Post-hoc* pairwise comparisons: The least significant difference (LSD) method was used for multiple comparisons. As this was an exploratory study, all outcomes were interpreted by combining main effects, interaction effects and clinical context.
4. Correlation analysis: Pearson correlation was used to assess associations between BMI and RV strain metrics, with all correlation coefficients (r) and matching 95% CIs calculated and reported.
5. Multivariable analysis: Multiple linear regression models were constructed to identify independent predictors of RV function. Each RV strain parameter was entered as the dependent variable, with BMI and T2DM status as independent variables. The models were adjusted for age, sex, estimated pulmonary artery systolic pressure (PASP), and left ventricular ejection fraction (LVEF). Standardized regression coefficients (β) were used to reflect the independent effect size of each variable.

A two-tailed P-value < 0.05 was considered statistically significant.

## Results

### Baseline information

There were no statistically significant differences in sex, age, heart rate, systolic blood pressure, diastolic blood pressure, or LVEF among the four groups (all P > 0.05), indicating well-balanced baseline characteristics across groups.

Fasting plasma glucose and HbA1c were significantly higher in the T2DM subgroups (groups C and D) compared with the non-diabetic subgroups (groups A and B) (all P < 0.05), with no significant difference between groups C and D. BMI was significantly higher in the overweight/obese subgroups (groups B and D) than in the normal weight subgroups with the same diabetes status (groups A and C) (all P < 0.05). The mean disease duration was 3.2 ± 1.5 months in Group C and 3.3 ± 1.6 months in Group D.

In addition to age matching, baseline balance was controlled for sex, BMI, lipid profiles, and LVEF during enrollment to maximize the balance of potential confounding factors across groups. The mean systolic blood pressure of all participants was < 110 mmHg, consistent with the study’s exclusion of hypertension and underlying cardiovascular disease (**Table 1**).

**Table 1:** Baseline clinical characteristics of participants across the four groups.

Variables	Group A (n=30)	Group B (n=31)	Group C (n=33)	Group D (n=34)	F/χ^2^	P
General characteristics						
Age (years)	41.47 ± 7.98	37.55 ± 7.52	41.94 ± 9.14	41.91 ± 8.78	2.145	0.098
Male (n, %)	17 (56.67)	15 (48.39)	16 (48.48)	19 (55.88)	0.867	0.833
Heart rate (bpm)	83.17 ± 14.42	80.94 ± 6.87	77.56 ± 7.75	76.52 ± 5.54	2.654	0.051
Systolic blood pressure (mmHg)	105.5 ± 7.2	106.81 ± 8.79	106.03 ± 8.61	104.24 ± 6.62	0.723	0.54
Diastolic blood pressure (mmHg)	69.93 ± 4.95	68.74 ± 5.39	69.50 ± 4.84	70.03 ± 4.54	0.489	0.691
BMI(kg/m^2^)	22.28 ± 1.66	28.25 ± 1.66 ^△^	22.8 ± 1.89	28.22 ± 1.3 ^△^	135.42	< 0.001
Biochemical parameters						
Fasting plasma glucose (mmol/L)	4.84 ± 0.45	4.81 ± 0.57	8.65 ± 1.10 ^*#^	8.61 ± 0.92 ^*#^	198.76	< 0.001
HbA1c (%)	4.95 ± 0.51	5.03 ± 0.49	7.02 ± 0.56 ^*#^	6.97 ± 0.57 ^*#^	152.34	< 0.001
Total cholesterol (mmol/L)	4.52 ± 0.68	4.65 ± 0.72	4.71 ± 0.65	4.68 ± 0.70	0.612	0.609
Triglycerides (mmol/L)	1.35 ± 0.42	1.42 ± 0.48	1.45 ± 0.51	1.48 ± 0.46	0.521	0.669
Left ventricular systolic function						
LVEF(%)	67.80 ± 2.48	66.06 ± 3.07^*^	66.03 ± 2.42 ^*^	66.56 ± 2.50	3.042	0.031
Left ventricular diastolic function						
Mitral E wave (cm/s)	84.67 ± 9.56	76.94 ± 8.52 ^*△^	74.06 ± 10.20 ^*^	67.97 ± 11.81 ^*#△^	14.828	< 0.001
Mitral A wave (cm/s)	45.53 ± 4.95	50.06 ± 4.55 ^*△^	56.06 ± 8.66 ^*#^	63.79 ± 12.79 ^*#△^	27.285	< 0.001
Mitral E/A	1.86 ± 0.14	1.55 ± 0.25 ^*△^	1.37 ± 0.33 ^*#^	1.13 ± 0.37 ^*#△^	35.998	< 0.001
Mitral e′(m/s)	0.15 ± 0.01	0.14 ± 0.01 ^*△^	0.15 ± 0.01 ^#^	0.14 ± 0.01 ^*△^	5.255	0.002
Mitral a′(m/s)	0.13 ± 0.01	0.13 ± 0.01	0.12 ± 0.01 ^*#^	0.12 ± 0.01 ^*^	6.521	< 0.001
Mitral e′/a′	1.15 ± 0.11	1.12 ± 0.12	1.25 ± 0.01 ^*#^	1.17 ± 0.15 ^#△^	8.319	< 0.001
Disease duration						
Disease duration (months)	–	–	3.2 ± 1.5	3.3 ± 1.6	0.231	0.822

*P < 0.05 compared with Group A; #P < 0.05 compared with Group B; △P < 0.05 compared with the normal weight subgroup with the same diabetes status.

### Conventional echocardiographic parameters

There were statistically significant differences in conventional RV functional parameters among the four groups (all P < 0.05). Compared with Group A, Group B, Group C and Group D showed gradual declines in E wave, RVFAC, and TAPSE, along with an increase in A wave and progressive abnormalities in E/A, E′/A′, and RV-MPI. The most pronounced alterations were observed in Group D.

Two-way analysis of variance (ANOVA) revealed significant main effects of both BMI and T2DM on these conventional parameters (all P < 0.05), as well as a significant interaction between the two factors (P < 0.05).Detailed results are presented in **Table 2**.

**Table 2:** Conventional RV echocardiographic parameters across the four groups (mean ± SD).

Variables	Group A (n=30)	Group B (n=31)	Group C (n=33)	Group D (n=34)	BMI main effect (P)	T2DM main effect (P)	Interaction effect (P)
RVFAC(%)	45.06 ± 1.24	44.62 ± 1.53	40.95 ± 1.02 ^*#^	37.55 ± 2.46 ^*#△^	< 0.001	< 0.001	< 0.001
TAPSE(mm)	24.82 ± 1.38	24.55 ± 1.75	22.98 ± 1.13 ^*#^	20.95 ± 1.79 ^*#△^	< 0.001	< 0.001	0.002
Tricuspid S′(cm/s)	13.31 ± 0.64	12.54 ± 0.60 ^△^	12.15 ± 0.55 ^*^	12.78 ± 1.18 ^*△^	0.807	0.002	< 0.001
RV-MPI	0.39 ± 0.03	0.38 ± 0.03	0.39 ± 0.02	0.35 ± 0.02 ^*#△^	< 0.001	<0.001	0.001

*P < 0.05 compared with Group A (LSD test); #P < 0.05 compared with Group B (LSD test); △P < 0.05 compared with the normal weight subgroup with the same diabetes status (LSD test).

### Two-dimensional speckle tracking imaging parameters

RV strain parameters measured by 2D-STI showed statistically significant differences among the four groups (all P < 0.05). Compared with Group A, RVFWS and SRe/SRa ratio declined gradually in Group B, C and D, with the most severe abnormalities in Group D.

Two-way ANOVA revealed significant interactions between BMI and T2DM for both RVFWS and the SRe/SRa ratio (all P < 0.001).Detailed results are presented in **Table 3**.

**Table 3:** RV free wall strain parameters assessed by 2D-STI (mean ± SD).

Variables	Group A (n=30)	Group B (n=31)	Group C (n=33)	Group D (n=34)	BMI main effect (P)	T2DM main effect (P)	Interaction effect (P)
RVFWS(%)	-25.75 ± 3.31	-21.36 ± 1.94 ^*^	-21.82 ± 1.39 ^*^	-20.42 ± 1.43 ^*#^	< 0.001	< 0.001	< 0.001
RVFWS-B(%)	-27.99 ± 3.46	-22.85 ± 2.89 ^*^	-22.60 ± 1.39 ^*^	-21.13 ± 2.24 ^*#^	< 0.001	< 0.001	< 0.001
RVFWS-M(%)	-25.35 ± 3.62	-21.04 ± 2.03 ^*^	-21.60 ± 1.66 ^*^	-20.34 ± 1.55 ^*#^	< 0.001	< 0.001	< 0.001
RVFWS-AP(%)	-23.92 ± 3.52	-20.19 ± 1.36 ^*^	-21.27 ± 2.32 ^*^	-19.77 ± 1.26 ^*#^	< 0.001	< 0.001	< 0.001
RVSRa(s^-1^)	1.28 ± 0.05	1.14 ± 0.11 ^*^	0.98 ± 0.03 ^*^	0.88 ± 0.17 ^*#^	< 0.001	< 0.001	< 0.001
RVSRe(s^-1^)	2.03 ± 0.10	1.64 ± 0.15 ^*^	1.57 ± 0.15 ^*^	1.05 ± 0.13 ^*#^	< 0.001	< 0.001	< 0.001
SRe/SRa	1.59 ± 0.09	1.45 ± 0.16 ^*^	1.40 ± 0.18 ^*^	1.21 ± 0.16 ^*#^	< 0.001	< 0.001	< 0.001

*P < 0.05 compared with Group A; #P < 0.05 compared with Groups B and C (LSD test for *post-hoc* multiple comparisons).

### Reproducibility of strain measurements

We randomly selected 20 ultrasound images for reproducibility testing. For intra-observer analysis, the intraclass correlation coefficient (ICC) was 0.941 and the coefficient of variation (CV) was 3.15% for RVFWS; the ICC was 0.934 and CV was 3.42% for SRe; the ICC was 0.927 and CV was 3.66% for SRa. For inter-observer analysis, the ICC was 0.916 and CV was 4.08% for RVFWS; the ICC was 0.905 and CV was 4.36% for SRe; the ICC was 0.899 and CV was 4.51% for SRa. All ICC values were > 0.90 and all CV values were < 5.0%, indicating good intra-observer and inter-observer agreement of the strain measurements.

### Correlation analysis between BMI and RV strain parameters

Pearson correlation analysis (**Table 4**) demonstrated that BMI was significantly and negatively correlated with RVFWS and all diastolic functional parameters (RVSRe, RVSRa, and SRe/SRa) in the overall population (all P < 0.001). Subgroup analysis showed that the negative correlation between BMI and RVFWS was consistent across all groups. However, the negative correlation with diastolic functional parameters was primarily observed in the T2DM population, whereas no significant correlation was found between BMI and SRe/SRa in the non-T2DM population.

**Table 4:** Pearson correlation analysis between BMI and RV strain parameters.

Strain parameters	Statistical parameters	Total population (n=128)	Group A (n=30)	Group B (n=31)	Group C (n=33)	Group D (n=34)
RVFWS	r	-0.728	—	- 0.763	- 0.775	- 0.646
	95%CI	(-0.799, -0.634)	—	(- 0.869, - 0.607)	(- 0.873, - 0.623)	(- 0.786, - 0.450)
	P	< 0.001	—	< 0.001	< 0.001	< 0.001
RVSRe	r	- 0.562	—	—	- 0.738	- 0.798
	95%CI	(- 0.669, -0.431)	—	—	(- 0.849, - 0.577)	(- 0.890, - 0.661)
	P	< 0.001	—	—	< 0.001	< 0.001
RVSRa	r	- 0.489	—	—	- 0.528	- 0.656
	95%CI	(- 0.607, - 0.348)	—	—	(- 0.697, - 0.303)	(- 0.794, - 0.464)
	P	< 0.001	—	—	< 0.01	< 0.001
SRe/SRa	r	- 0.587	—	—	-0.749	- 0.789
	95%CI	(- 0.690, - 0.461)	—	—	(-0.856, -0.596)	(- 0.884, - 0.645)
	P	< 0.001	—	—	<0.001	< 0.001

r, Pearson correlation coefficient; 95% CI, 95% confidence interval of correlation coefficient. “—” indicates non-significant Pearson correlation (P ≥ 0.05), for which specific values are not displayed. P < 0.05 was defined as statistically significant.

### Multivariable analysis of the impact of BMI and T2DM on RV strain parameters

Multiple linear regression models were built for all participants, with each RV strain parameter entered as the dependent variable and BMI and T2DM status as independent variables. All models were adjusted for age, sex, PASP, and LVEF.

As shown in **Table 5**, after adjustment for confounders, both BMI and T2DM status were independently associated with RV systolic and diastolic dysfunction (all P < 0.001). BMI exerted a stronger independent negative effect on RVFWS (β = − 0.701), whereas T2DM demonstrated more pronounced independent negative effects on RVSRe and RVSRa (β = −0.674 and −0.720, respectively). These findings suggest that obesity primarily impairs RV systolic function, while diabetes has a more prominent detrimental effect on RV diastolic function.

**Table 5:** Multiple linear regression analysis of independent predictors of RV strain parameters (adjusted for age, sex, PASP, and LVEF).

Dependent Var	Predictor	Unstandardized B	SE	95% CI lower	95% CI upper	Standardized β	P
RVFWS	BMI	-0.312	0.044	-0.399	-0.225	-0.701	< 0.001
	T2DM status	-1.268	0.231	-1.724	-0.812	-0.487	< 0.001
RVSRe	BMI	-0.027	0.004	-0.035	-0.019	-0.573	< 0.001
	T2DM status	-0.163	0.028	-0.218	-0.108	-0.674	< 0.001
RVSRa	BMI	-0.011	0.003	-0.017	-0.005	-0.276	< 0.001
	T2DM status	-0.142	0.021	-0.183	-0.101	-0.720	< 0.001
SRe/SRa	BMI	-0.018	0.003	-0.024	-0.012	-0.426	< 0.001
	T2DM status	-0.115	0.022	-0.158	-0.072	-0.479	< 0.001

T2DM status: 1 = patients with type 2 diabetes mellitus, 0 = non-diabetic subjects; B = unstandardized regression coefficient; SE = standard error; CI = confidence interval; β = standardized regression coefficient. P < 0.05 indicates statistical significance.

## Discussion

We rigorously excluded confounders including hypertension and pulmonary disorders. All newly enrolled patients had early-stage mild metabolic disorders with uniformly low baseline blood pressure, eliminating confounding effects from chronic pressure overload on the right ventricle. On this basis, we identified subclinical systolic and diastolic RV dysfunction in patients with newly diagnosed T2DM. Multivariate analysis further demonstrated that both BMI and T2DM are independent influencing factors for reduced RV function, yet they act through distinct mechanisms: obesity shows a stronger correlation with impaired RV systolic function, whereas T2DM correlates more strongly with impaired RV diastolic function.

We also observed that left ventricular diastolic function progressively declined with increasing BMI and the presence of T2DM, consistent with the trend of RV functional changes, which aligns with the theory of ventricular interdependence (15–17). The left and right ventricles share the interventricular septum, pericardium, and muscle fibers, and left ventricular diastolic dysfunction can affect RV filling through mechanisms such as septal shift and pericardial constraint. However, after adjustment for left ventricular functional parameters including LVEF, BMI and T2DM still exert significant independent effects on RV function. This indicates that obesity-related factors such as insulin resistance, chronic systemic inflammation and myocardial metabolic disorders may participate in the progression of myocardial functional abnormalities in diabetic patients, and the above changes may serve as the potential mechanisms linking the two metabolic conditions to abnormal RV function.

In this study, confounding factors such as hypertension, cardiovascular disease, and bronchopulmonary disease were rigorously excluded. The results showed that compared with non-diabetic controls, patients with T2DM had significantly lower RVFWS and SRe/SRa ratios, and conventional ultrasound parameters (E/A, RVFAC, TAPSE) were also abnormal; this indicates subclinical RV dysfunction develops at the early stage of T2DM. 2D-STI is more sensitive than conventional echocardiography for detecting subtle myocardial deformation. RVFWS and SRe/SRa can act as imaging biomarkers to identify early RV functional abnormalities in diabetic populations.

The present study found a significant interaction between BMI and T2DM on RV functional decline. The reductions in RVFWS and SRe/SRa were more pronounced in T2DM patients with overweight/obesity compared with normal-weight diabetic patients, and BMI was an independent predictor of RV functional parameters. This association may be related to increased metabolic load, insulin resistance, systemic inflammatory response, and ectopic myocardial fat deposition, which is consistent with conclusions from previous studies (18, 19). Notably, all T2DM patients included in this study were newly diagnosed and treatment-naïve, which excluded the confounding effects of disease duration and antidiabetic medications and allowed for a clearer assessment of the effect of T2DM itself on RV function.

All T2DM patients included in this study had early-stage disease with HbA1c ≤ 9%, and those with left ventricular dysfunction, coronary heart disease, or other complications that might affect RV function were excluded, maximizing control of confounding factors in the study design. This was a single-center cross-sectional exploratory study. Previous cardiac magnetic resonance (CMR) studies have suggested that RV strain in T2DM patients is associated with disease duration rather than BMI, and discrepancies in conclusions may be related to differences in imaging modalities, disease severity, and inclusion criteria (20, 21). Notably, for patients with preserved ejection fraction, 2D-STI has higher sensitivity for detecting early myocardial strain abnormalities, which explains why subclinical dysfunction could be detected in the present study.

The results of this study highlight the clinical value of advanced echocardiographic techniques in the detection of early myocardial dysfunction in high-risk populations. RVFWS reflects global longitudinal RV systolic function, SRe and SRa assess early and late diastolic filling, respectively, and the SRe/SRa ratio is a comprehensive indicator of RV diastolic function. These strain parameters reflect RV functional abnormalities more comprehensively and sensitively than traditional indicators such as TAPSE and RVFAC. Therefore, incorporating strain analysis into routine echocardiographic evaluation of T2DM patients—especially those with elevated BMI—may help improve early diagnostic rates and guide timely intervention.

### Limitations

First, this was a single-center study with a relatively small sample size, which may limit the generalizability of the results. Second, patients with poor glycemic control (HbA1c > 9%) were not included, which may have underestimated the impact of severe hyperglycemia on RV function. Third, the cross-sectional design cannot establish causal relationships or longitudinal progression. Fourth, biomarkers such as inflammatory cytokines and insulin resistance indices were not measured, so the specific molecular mechanisms cannot be fully elucidated. Fifth, two-way ANOVA only identified a statistical interaction between BMI and T2DM. We did not test biomarkers such as inflammatory cytokines, insulin resistance indices and myocardial fat deposition, so the molecular mechanisms mediating their combined cardiac damage remain unelucidated and warrant further basic research and long-term cohort validation. Sixth, image acquisition frame rates spanned 50–70 Hz (mean 62.4 ± 5.8 Hz), complying with the ASE minimum standard for RV strain analysis. However, higher frame settings can boost temporal resolution of diastolic strain waveforms; future protocols will adopt frame rates ≥75 Hz to mitigate the risk of underestimated diastolic strain peaks.

## Conclusions

Elevated BMI and T2DM are both independently linked to RV myocardial injury, and the two conditions differ in their predominant impacts on ventricular function: obesity shows a stronger correlation with reduced RV systolic function, while T2DM is more prominently linked to impaired RV diastolic function. Abnormal left ventricular function can aggravate RV filling dysfunction via ventricular interaction. Metabolic alterations linked to obesity and T2DM may correlate with subclinical RV myocardial injury via multiple pathological pathways. Clinically, early screening for RV function should be emphasized in overweight/obese patients with T2DM, with special attention paid to diastolic parameters. This approach facilitates the early detection of subclinical RV dysfunction and provides imaging evidence for the early intervention against cardiometabolic complications.

## Data Availability

The raw data supporting the conclusions of this article will be made available by the authors, without undue reservation.
